# Fourteen-analyser high-resolution hard X-ray emission spectrometer at I20 beamline at Diamond Light Source

**DOI:** 10.1107/S1600577526001736

**Published:** 2026-03-25

**Authors:** Shusaku Hayama, David Butler, Leo Cahill, J. Frederick W. Mosselmans, Steven Richards, Silvia Ramos, Iain Hall, Huw Shorthouse, Sofia Diaz-Moreno

**Affiliations:** a Diamond Light Source Ltd Harwell Science and Innovation Campus Didcot OxfordshireOX11 0DE United Kingdom; b School of Physics and Astronomy University of Kent CanterburyCT2 7NH United Kingdom; Advanced Photon Source, USA

**Keywords:** X-ray emission spectrometer, valence-to-core emission spectroscopy, beamline endstation

## Abstract

We report the technical design, implementation and operation of a newly developed X-ray emission spectrometer on the I20 beamline at Diamond Light Source. The spectrometer consists of 14 crystal analysers arranged in an up–down configuration, allowing for operation in one- or two-colour acquisition modes. Since beginning operations in 2023, the spectrometer has substantially enhanced the capability of the beamline to perform X-ray emission spectroscopy (XES) and has demonstrated high reliability with minimal operational issues during user experiments. The latter achievement is particularly significant given the complexity of the instrument, and the difficulty of maintaining the Rowland condition when scanning the energy. We show that the spectrometer can effectively measure spectra in two-colour mode and is capable of detecting weak valence-to-core emission features with a significantly improved signal-to-background ratio. We also present data taken using a newly developed quick-scanning XES acquisition mode, which enables data collection in seconds rather than minutes. This mode opens up possibilities for time-resolved studies and investigating radiation-sensitive materials.

## Abstract 2

A high-efficiency 14-analyser X-ray emission spectrometer that offers novel capabilities has been developed for I20-Scanning at Diamond Light Source.

## 1. Introduction

X-ray emission spectroscopy (XES) detects secondary photons emitted when electrons from higher energy levels fill the core holes created by the X-ray absorption process. The electrons that decay can originate from either core or valence levels, allowing different occupied states to be probed by tuning to various emission energies. For example, in the case of 3*d* transition metals, this could be *K*α (2*p* → 1*s*), *K*β (3*p* → 1*s*) or *K*β_2,5_ (4*p*, 3*d* character → 1*s*) emission lines. The latter is a result of transitions of electrons in valence orbitals and provides information about the nature of the interaction between the valence and ligand orbitals. XES is performed by scanning the spectrometer over the emission line of interest while keeping the incident energy at a fixed value well above the absorption edge, typically between 100 and 200 eV. XES can also be performed in resonance mode when the incident energy is tuned to a resonant feature in the absorption edge. Resonant X-ray emission spectroscopy (RXES) usually enhances the spectral features in the emission spectrum, and the difference in the energy between the incident and emitted photons can reveal different final states which are not accessible with conventional X-ray absorption spectroscopy (XAS). Therefore, XES/RXES in conjunction with XAS allow for more precise mapping of the local electronic structures and can provide detailed insights into the chemical environment of the element of interest in the material.

Over the past two decades, XES and RXES have gained significant popularity and have found applications across a wide range of scientific disciplines, including materials science, chemistry, catalysis and biological science (de Groot, 2001 ▸; Glatzel & Bergmann, 2005 ▸; Bauer, 2014 ▸; Cutsail & DeBeer, 2022 ▸; Gallo & Glatzel, 2014 ▸; Martinie *et al.*, 2018 ▸). This growth is primarily driven by advancements and widespread availability of high-resolution XES spectrometers at synchrotron radiation facilities that enable more efficient detection of photons emitted from samples (Glatzel *et al.*, 2021 ▸; Kleymenov *et al.*, 2011 ▸; Sokaras *et al.*, 2013 ▸; Tayal *et al.*, 2024 ▸). These spectrometers offer sufficient energy resolution to resolve the natural line widths of emission lines whilst maximizing the detection efficiency. A widely used method for conducting XES involves using multiple bent Bragg optics, known as analysers, to select and focus the emitted photons from the sample. These analysers are typically arranged in either point-to-point Rowland or von Hamos geometries. The Rowland geometry is often preferred for its ability to suppress background signals and offer high-energy resolution. In contrast, the von Hamos geometry allows for energy-dispersive detection of the XES spectrum, making it particularly advantageous for applications that require rapid data acquisition (Zimmermann *et al.*, 2020 ▸; Szlachetko *et al.*, 2012 ▸).

I20-Scanning has been operating with an XES spectrometer equipped with three 100 mm-diameter spherical analyser crystals since 2016 (Hayama *et al.*, 2021 ▸). The spectrometer was designed in-house and was based on a point-to-point Johann geometry with a 1 m Rowland circle working in the vertical direction. Each analyser and detector were mounted on a dedicated motorized platform and designed to precisely follow the Rowland coordinates when changing the energy of the spectrometer. Many innovative experiments have been successfully conducted using this three-analyser system (see https://www.diamond.ac.uk/Instruments/Spectroscopy/I20/Publications.html). In particular, the instrument proved to be highly effective for high-energy-resolution fluorescence detection (HERFD) XAS, thanks to the four-bounce monochromator installed in the beamline (Hayama *et al.*, 2018 ▸) which provides highly stable incident X-rays with the optimized bandpass of Δ*E*_i_/*E*_i_ = 10^−4^ (where *E*_0i_ is the incident energy) at the sample position. However, with only three crystal analysers, the spectrometer could only capture a small fraction of the photons emitted by the sample (one 100 mm-diameter analyser will capture ∼0.063% of the total solid angle at 1 m from the sample), and this has often restricted experiments that we could conduct on the beamline. To address this limitation and support the user community in performing increasingly complex XES experiments, we have designed and built a new spectrometer with 14 crystal analysers. In this paper, we introduce this new emission spectrometer developed for the I20-Scanning beamline at Diamond Light Source. The new instrument retains the same 1 m Rowland geometry and Johann configuration to maintain the energy resolution achieved with the three-crystal analyser system. Additionally, it features a unique layout that allows simultaneous measurement of two different emission lines. We demonstrate the main capabilities of the spectrometer by presenting L3-HERFD-XAS, valence-to-core (VtC) and resonance VtC emission measurements taken from a series of tungsten standard samples. With 14 crystal analysers, the spectrometer is currently one of the largest point-to-point XES spectrometers in the world, and numerous experiments have already been successfully conducted to take advantage of new opportunities offered by the spectrometer.

## 2. Spectrometer design and alignment

**Figure 1:**
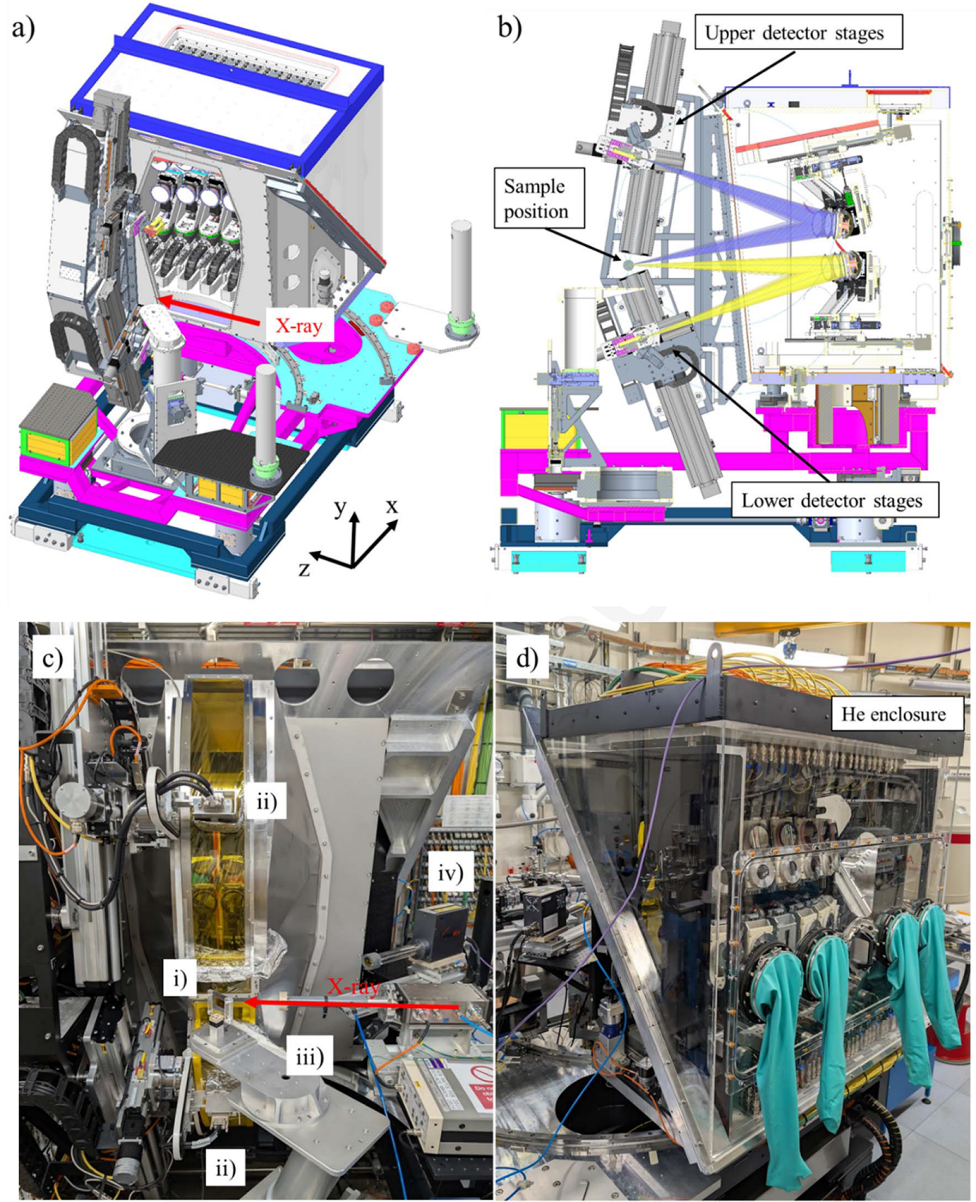
Overview of the I20 XES 14-crystal analyser spectrometer. (*a*) Top-side schematic view of the whole spectrometer. (*b*) Cross section view showing the emitted rays from the sample to the upper (purple) and lower (yellow) analysers, and then to the detectors. (*c*) Photograph around the sample area: (i) sample, (ii) Medipix detectors, (iii) sample stages, (iv) four-element Si drift detector. (*d*) Photograph taken from the back of the spectrometer. Additional sets of analysers are stored behind the analyser stages in a Perspex helium enclosure.

The mechanical design of the I20 spectrometer is illustrated in Figs. 1 ▸(*a*), 1 ▸(*b*). The overall design of the spectrometer was driven by two key factors. First, there was a need to minimize the horizontal collection angle of the instrument. The more crystal analysers we include in the same horizontal plane, the larger the horizontal opening required at the sample position will be. This will significantly restrict the types of sample environments that can be used with the spectrometer, and/or lead to shading of the more extreme crystal analysers. To address this, we decided early in the design process to restrict the total horizontal collection angle to below ±30°, which consequently limited the number of crystal analysers that could be arranged in a single row to seven, considering that each analyser is 100 mm in diameter. The second consideration was to enhance the flexibility of the instrument by incorporating two independent Rowland geometries. This led to having two rows of crystal analysers, with one row focusing on a detector placed above the sample position and the other focusing on a detector placed below the sample position, in an up-and-down geometry. This up-and-down geometry allows the two rows to operate individually and the instrument to be configured for increased signal collection from a single emission line (one-colour mode) or for simultaneous measurement of two emission lines (two-colour mode) within a single experimental session. The use of two different detectors, each dedicated to collecting the signal from a particular row, ensures the Rowland condition is always satisfied for both the upper and lower rows. The angular range of the spectrometer is from 65° to 87° for the upper row and from 65° to 85° for the lower row, covering the energy range from 5 keV to 18 keV, with a wide selection of analyser cuts available at Diamond Light Source (Hayama *et al.*, 2021 ▸).

The spectrometer consists of several key mechanical components: analyser platforms, detector platforms, a base and a helium enclosure, and each of these components will be discussed in detail below.

One of the crucial technical challenges of a multiple-crystal analyser system for a point-to-point spectrometer is to design a platform that can accurately position the centre of each crystal analyser on the Rowland coordinates as the energy of the spectrometer changes. For this spectrometer, we opted to follow the Rowland circles using the same methodology implemented in our previous three-crystal analyser systems (Hayama *et al.*, 2021 ▸). In this approach, the central crystal analyser serves as a master and is translated along the *x* axis, while the Bragg angle is selected by changing the pitch angle. The other ‘side’ crystal analysers are off-centre and must be moved in *x*, yaw, *y* and pitch directions to stay on their own Rowland circles, and to direct the diffracted secondary photons onto the same point on a detector. Therefore, in order to change the energy of the seven crystal analysers in each row, a total of 26 axes need to be accurately adjusted to select the energy of the emitted photons.

**Figure 2:**
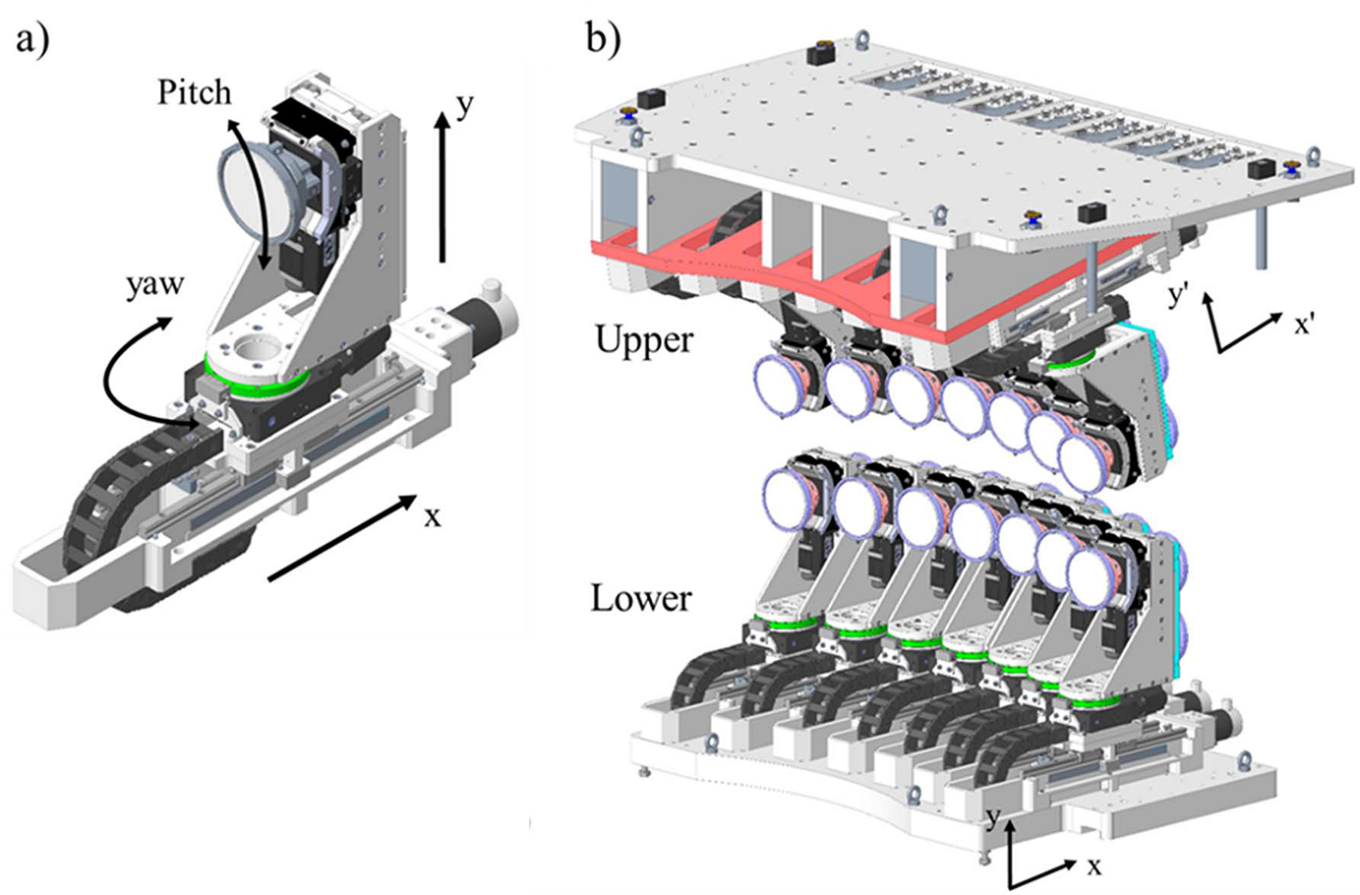
Schematic view of the upper and lower crystal analyser stages. (*a*) Design of the individual crystal analyser platform. All 14 stacks have identical configurations with *x*, *y,* yaw and pitch movements. (*b*) The upper plate is inclined 11° with respect to the *x* axis. Both crystal analyser platforms are aligned to point towards the sample position.

Each crystal analyser is mounted on an identical stage stack that includes motorized stages for *x*, yaw, *y* and pitch adjustments [Fig. 2 ▸(*a*)]. The *x* stages are designed in-house and feature cable management underneath to minimize the spacing between adjacent crystal analyser stacks to just 130 mm. Thanks to this compact design, the maximum horizontal viewing angle of the spectrometer is only ±29.4° for seven crystal analysers of 100 mm diameter. The *y* stages are also developed in-house and use a PI high-resolution actuator (Physik Instrumente L-220.50SG). The yaw and pitch stages are sourced from Newport (URS100BPP, BGS80PP). All stages are equipped with encoders, with the *x* and yaw stages fitted with absolute RESOLUTE encoders from Renishaw plc. With the absolute encoders, the position feedback is done without the need for homing, considerably reducing the risk of collision with adjacent stages. The crystal analyser stages are specified to provide sufficient resolution and repeatability to allow for a step size of 0.1 eV in the working range of the spectrometer. It is important to note that non-standard RESOLUTE readheads were used for our analyser stages as the standard version uses a microelectromechanical (MEMS) oscillator, which is not suitable for use in helium environments.

The seven-crystal analyser stacks are mounted on a robust solid platform, with the lower row positioned to align the central crystal analyser at the same height as the sample position (*x*, *z*, *y*) = (0, 0, 0). The *x* axis of the lower row platform is aligned horizontally to match the Cartesian coordinate system of the beamline. In contrast, the upper crystal analyser platform is fixed at an 11° incline relative to the *x* axis of the beamline and installed with the crystal analyser stacks hung upside down in comparison with those in the lower row [Fig. 2 ▸(*b*)]. This design allows the upper and lower crystal analysers to be directly stacked on top of each other with a minimum vertical spacing between the two rows. This layout helps to keep the vertical collection angle of the instrument between −6° and 17°.

Although the upper and lower rows do not share the same Cartesian coordinates, the crystal analysers in both rows maintain an identical geometrical layout relative to the sample position within their respective frames. For instance, the *x* and *y* stages in both rows move the crystal analysers purely along their *x* axis and *y* axis, and the pitch and yaw stages have no parasitic motion [Fig. 2 ▸(*a*)]. This configuration allows the same crystal analyser stacks to be used for both rows, and we can move the crystal analysers using the same coordinated system to follow the Rowland geometry and change the energy of the spectrometer. This is crucial as the upper and lower crystal analyser stages can have the same specifications and help to achieve a similar energy resolution with both rows.

**Figure 3:**
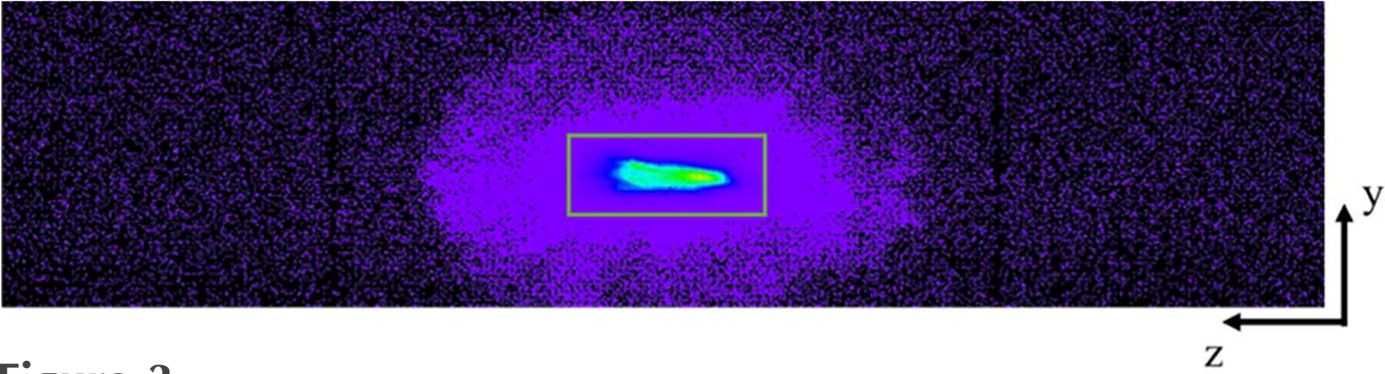
An example of the emission spots captured by the Medipix detector. The image was taken with seven Si(642) analysers focusing on the same spot to measure the Zn *K*α line at the spectrometer angle of 81.4°. The green box (region of interest, ROI) indicates the area used to sum the emission signal. Once the spectrometer is aligned, the ROI is fixed, as the focal spots remain consistent during measurements. The ROI is also used to align the sample to the origin of the spectrometer by adjusting its position until the emission spots are placed within the green box.

The role of the detector stages is to position the detector on the independent Rowland circles and to track the focal spots of the analysers. The trajectory that the detector must follow can be expressed in Cartesian coordinates 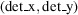 as
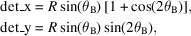
where *θ*_B_ is the Bragg/reflection angle with the origin (0, 0, 0) at the sample position. In our set-up [Fig. 1 ▸(*b*)], we have chosen to tilt the vertical stages of the detector away from the vertical plane, with the lower vertical stage tilted 20° counterclockwise, while the upper vertical stage is tilted 9° towards the clockwise direction. This tilting arrangement ensures that the upper and lower detector stages are mirror images of each other with the same 20° of rotation with respect to the Cartesian axis of the perspective crystal analyser platform. The vertical stages are tilted to minimize the travel range required for the horizontal stage, which in turn reduces the weight the vertical stage needs to support. The movements required for both the vertical and horizontal stages in this tilted layout can be expressed as follows:
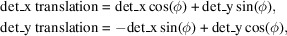
where ϕ is the tilting angle, −20°. Each upper and lower row is equipped with a Medipix-Merlin photon-counting pixel detector (see https://quantumdetectors.com/products/merlinx/). The Medipix is a high-performance X-ray imaging detector developed at Diamond Light Source, and the spectrometer is fitted with a 1 × 4 element version that provides an active area of 56 mm × 14 mm (width × height) with 1024 × 256 pixels. A large area detector is essential for aligning the emission spots from the analysers and checking the focus quality and the radius of curvature of each analyser. The Medipix detector can operate with either a 12-bit or 24-bit dynamic range per pixel, and it has a signal processing feature that allows for the selection of a specific rectangular region to sum the counts for measurements. This feature is used to optimize the signal-to-background ratio and only selects the diffracted beams from the crystal analysers that achieve the best spectral resolution. An example of the emission spot from seven analysers focused on the same space is shown in Fig. 3 ▸. The images captured by the detectors during the measurements are available for diagnosing issues or post-processing to enhance data quality (Sier *et al.*, 2025 ▸).

The base of the spectrometer is supported with four independent vertical jacks (Instrument Design Technology, UK, 1 tonne version with a travel range of 50 mm) and these jacks are grouted on the floor of the experimental hutch of I20-Scanning. All the spectrometer components, including the sample stages, are securely attached to this structure to ensure that the alignment of the analyser and detector stages relative to the sample position is maintained. The jacks can lift the entire spectrometer, and a motorized heavy-duty rail is also incorporated for translating the spectrometer perpendicular to the incoming beam. As the jacks are independent, small adjustments of the base in pitch and roll are also possible. Mounted on top of this base is a rotational yaw stage, which allows the whole spectrometer to be rotated between 50° and 90° to the incident beam. While this yaw motion is not strictly necessary, it significantly enhances the flexibility of the spectrometer by making it possible to accept a wider variety of sample environments by avoiding a fixed 90° geometry.

The absorption coefficient of air is not negligible in the working energy range of the spectrometer (5 keV to 18 keV). For example, only about 30% of photons will be transmitted through 200 cm of air when working at 10 keV. It is therefore essential to minimize the air paths between the sample, crystal analysers and detectors. We have opted to enclose the entire crystal analyser assembly under helium in a Perspex enclosure [Fig. 1 ▸(*d*)]. Although this option makes the change of crystal analysers more challenging, it minimizes the air path between the sample and the crystal analysers while reducing the number of windows the emitted photons must go through to reach the detector. The helium enclosure has been designed to allow the crystal analysers to be loaded from the back of the spectrometer using a load lock system. Once loaded, the crystal analysers can be mounted on the stages using gloves without breaking the helium atmosphere. The enclosure can store up to five sets of seven crystal analysers at one time to speed up the alignment process. The helium level inside the enclosure is monitored with a helium sensor and is typically filled with less than 1 l min^−1^ to achieve over 90% helium concentration inside.

The 14-crystal analyser spectrometer was installed and aligned following a similar methodology developed for the previous spectrometer and described by Hayama *et al.* (2021 ▸). Once the spectrometer was fully assembled in the experimental hutch, a laser tracker was used to align and establish the locations of the crystal analysers and detector in relation to the sample position of the spectrometer with an accuracy of ±50 µm. This process identified the calibration offsets required for each crystal analyser and detector stage to position them correctly within the three-dimensional space of the spectrometer. Special attention was paid to ensuring that when the *x* and *y* stages of the crystal analysers were set to 1000 mm and 0 mm, respectively, the centre of the crystal analyser was accurately set to the correct height and translated 1000 mm relative to the sample position (*x*, *y* = 0, 0). The laser tracker was also used to verify the trajectory of the crystal analysers and detectors in their operational range to ensure each stage was moving linearly within the frame of the spectrometer.

At the start of each experiment, we follow a series of steps to ensure the energy of the spectrometer is calibrated to measure the selected emission line. First, we align the origin of the spectrometer with the incoming beam by placing a 0.8 mm pinhole upstream of the sample position. This aperture has been pre-surveyed to ensure it is at the same height and aligned in the *x* direction to identify the origin of the spectrometer, 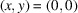. The base of the spectrometer is then adjusted in both the *x* and *y* directions until the incident beam passes through the centre of the pinhole. Next, we set the Rowland circle, R, to the value previously found to attain the best focus and energy resolution for each set of crystal analysers and set the Miller indices (*hkl*) to the required values to measure the selected emission line. We then correct for the miscut of each crystal analyser crystal to find the diffracted spot on the Medipix detector. This alignment is done separately for each crystal analyser by tweaking the pitch and yaw stages of the crystal analyser stack to put the diffracted spot at the centre of the detector (Fig. 3 ▸). As we have opted to mount our crystal analyser permanently on a dedicated kinematic holder, the offset values found from the previous alignment provide a good starting position and only minor adjustments are required to achieve this step. Once the spot is spatially aligned, we measure the emission line of interest (or elastically scattered peak) and calibrate the peak of the emission line to the tabulated value. These geometrical and energy alignment procedures are semi-automated using a bespoke script to improve efficiency. Finally, once all the crystal analysers are separately aligned, we measure the emission spectrum using all the crystal analysers and the combined energy resolution of the measurements is checked by measuring an elastic peak with the monochromator set to the emission energy. It is important to note that the quality of the crystal analyser can differ significantly from one to another, and the use of multiple crystal analysers can impact the overall energy resolution of the measurement. Therefore, we decide to include or exclude a crystal analyser on a case-by-case basis, considering the efficiency and energy resolution required by the experiment. For example, when measuring weak but broad emission lines, such as VtC emissions, we include all available analysers to maximize the collected signal. However, when measuring HERFD-XANES, we may choose to exclude some analysers if the overall energy resolution is degraded by more than ∼20%. The incident beam size of I20-Scanning is fixed at 400 µm (h) × 300 µm (v) FWHM and is not altered to adjust the energy resolution of the measurements.

## 3. Spectrometer capabilities

Tungsten is a third-row transition metal with four electrons in its 5*d* orbitals, and it can form a wide range of compounds that have been frequently studied using X-ray spectroscopy (Yamazoe *et al.*, 2008 ▸; Jayarathne *et al.*, 2014 ▸; Wach *et al.*, 2020 ▸; Smolentsev *et al.*, 2011 ▸). Here, we demonstrate the diverse capabilities of the I20 XES spectrometer by measuring four reference tungsten compounds, WC, W_2_C, WO_2_ and WO_3_, using different acquisition modes developed for the spectrometer. The selected carbide and oxide compounds also illustrate how *L*_3_ HERFD-XANES and XES spectra are sensitive to changes in the electronic states and chemical environments surrounding the metal ions. All spectra presented here were collected using a user-friendly GUI-based interface developed for the spectrometer.

### 3.1. Non-resonant X-ray emission spectroscopy

**Figure 4:**
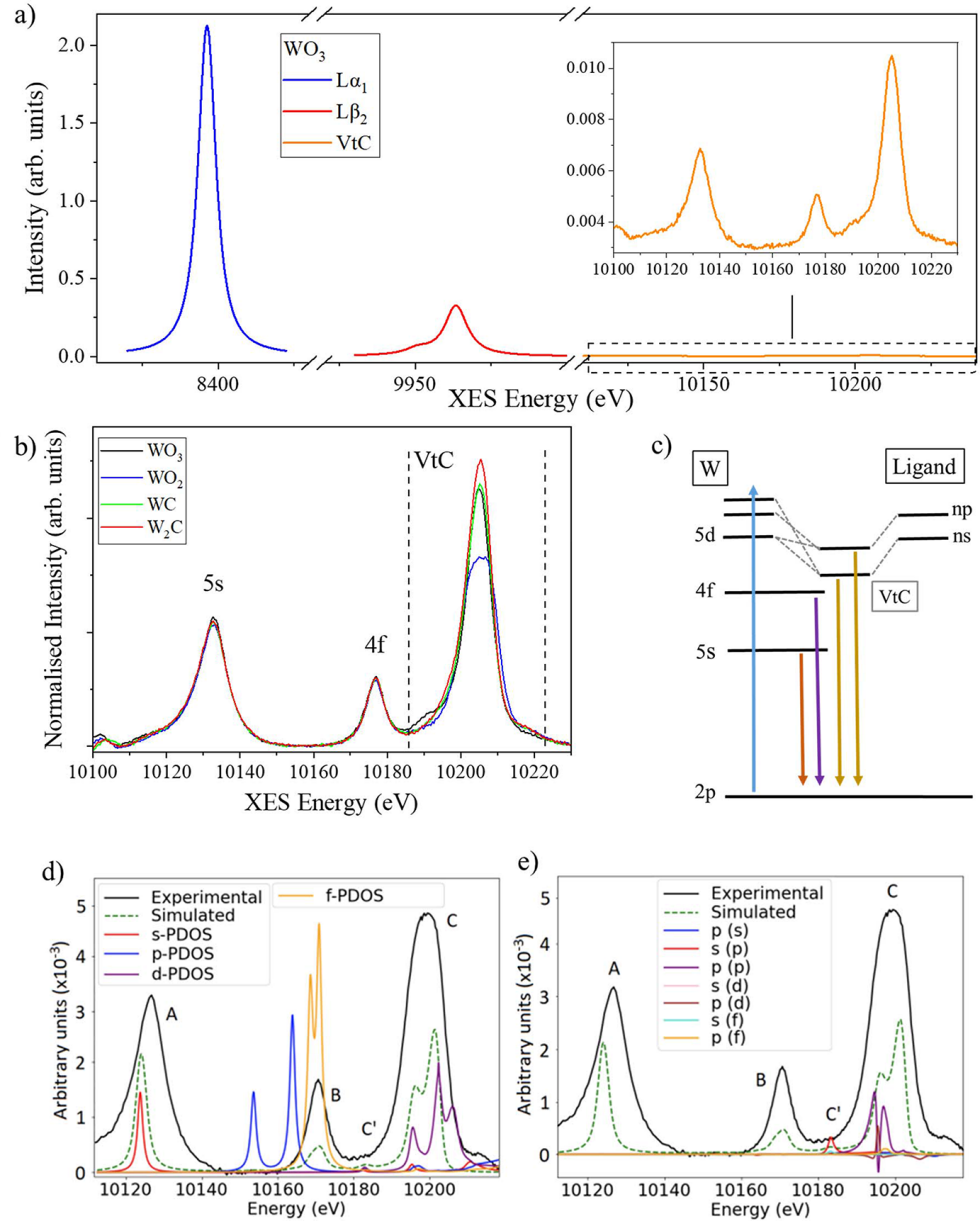
(*a*) Comparison of intensities between *L*α_1,_*L*β_2_ and VtC lines measured from the same sample using Si(444) and Si(555) crystal analysers. (*b*) VtC spectra taken from concentrated WO_3_, WO_2_, WC and W_2_C pellets in one-colour mode. The spectra have been normalized to the height of the core-to-core *L*β_2_ line after background subtraction. (*c*) Proposed emission diagram indicating possible origins of the VtC emission lines. The blue arrow indicates the X-ray absorption process. (*d*, *e*) Simulated DOS and ligand–metal orbital overlap for WO_2_, respectively. These preliminary calculations aid in the identification of novel emission lines (black line). Comparison with the DOS allows the measured features labelled A, B and C to be assigned to 5*s* (red), 4*f* (yellow) and 5*d* (purple) occupied states, respectively. As expected, panel (*e*) indicates a significant overlap with ligand orbitals in the valence region (C), whereas no pronounced peaks or orbital overlap are observed in the 5*s* and 4*f* regions (A and B).

Non-resonant X-ray emission spectroscopy is performed by scanning the spectrometer over the emission line of interest while keeping the incident energy at a fixed value well above the absorption edge, typically between 100 and 200 eV. Fig. 4 ▸(*a*) shows an example of an emission spectrum of WO_3_ at the W *L* edge, covering the emission range from 8300 eV to 10240 eV.

As seen in Figs. 4 ▸(*a*) and 5 ▸, the intensity in the VtC region is approximately 200 times weaker than the *L*α_1_ line and 30 times weaker than the *L*β_2_ line. Despite notable advancements in utilizing the VtC region in recent years, its application remains technically challenging due to its intrinsically lower intensity compared with deeper core-to-core transitions. The new I20 spectrometer significantly enhances detection efficiency and it enables better discrimination of weak emission features from the background level. This improvement directly addresses one of the main limitations of VtC XES, opening up new possibilities for applying the technique to a broader range of systems. Fig. 4 ▸(*b*) shows the normalized VtC spectra measured from concentrated WO_3_, WO_2_, WC and W_2_C pellets. Each spectrum was collected across the energy range between 10100 eV and 10240 eV using an energy step of 0.4 eV, with 3 s acquisition time per data point. The spectra are the result of the signal collected from 14 Si(555) crystal analysers, and three consecutive scans were merged to improve the signal-to-noise ratio of the measurements. The energy of the spectrometer was calibrated by measuring an elastic line with the monochromator set to the W *L*_3_ edge (10207 eV).

To assist in identifying the origins of the spectral features shown in Fig. 4 ▸(*b*), the partial density of states (DOS) and orbital overlap were calculated using the *FDMNES* code [Figs. 4 ▸(*d*), 4 ▸(*e*)] (Richards, 2022 ▸; Bunău & Joly, 2009 ▸). Similar calculations performed using the *FEFF9.6* code on tungsten metal and WO_3_ have been reported elsewhere (Wach *et al.*, 2020 ▸). As expected, the DOS calculations for all the reference samples show that the feature at 10133 eV is exclusively *s* in character, while the feature at 10177 eV is mostly *f* in character (Richards, 2022 ▸). This confirms that these features originate from the decay of electrons from shallow metal core orbitals, 5*s* and 4*f*, to fill the 2*p* core hole. The integrated intensity of these features remains constant for the reference compounds studied, as the 5*s* and 4*f* orbitals are fully occupied and not directly involved in bonding. It is interesting to note that the DOS shows a feature with a significant *p* character just below 10177 eV, but this is not reproduced in the experimental spectra as the emission from the 5*p* orbitals is not allowed under the symmetry conditions. In contrast to the core-to-core lines, the features between 10185 eV and 10220 eV show significant variations in intensity and spectral shape among the measured reference samples, notably WO_3_ displaying a distinct feature at 10190 eV. The DOS calculations in this region indicate the presence of 5*d* orbitals from tungsten with some additional contributions from the *s* and *p* orbitals of ligands. This suggests orbitals in this region are hybridized with both ligand and metal character, similar to what is observed in the *K*-edge VtC (Bauer, 2014 ▸; Cutsail & DeBeer, 2022 ▸). We also note that the intensity of the peak at 10205 eV in WO_3_, where all the 5*d* electrons (W^+6^, 5*d*^0^6*s*^0^) participate in bonding to oxygen atoms, is greater than in WO_2_ (W^+4^) but similar to that in WC (W^+4^). This shows that the intensity of this feature is not directly correlated with the 5*d* occupancy (formal oxidation state) of the metal but depends more on the nature and type of orbital interactions with the ligands. Although further simulation work is required to fully characterize the valence orbitals in these materials, we can deduce a simple transition diagram to illustrate the primary origins of the observed spectral features in this region [Fig. 4 ▸(*c*)].

**Figure 5:**
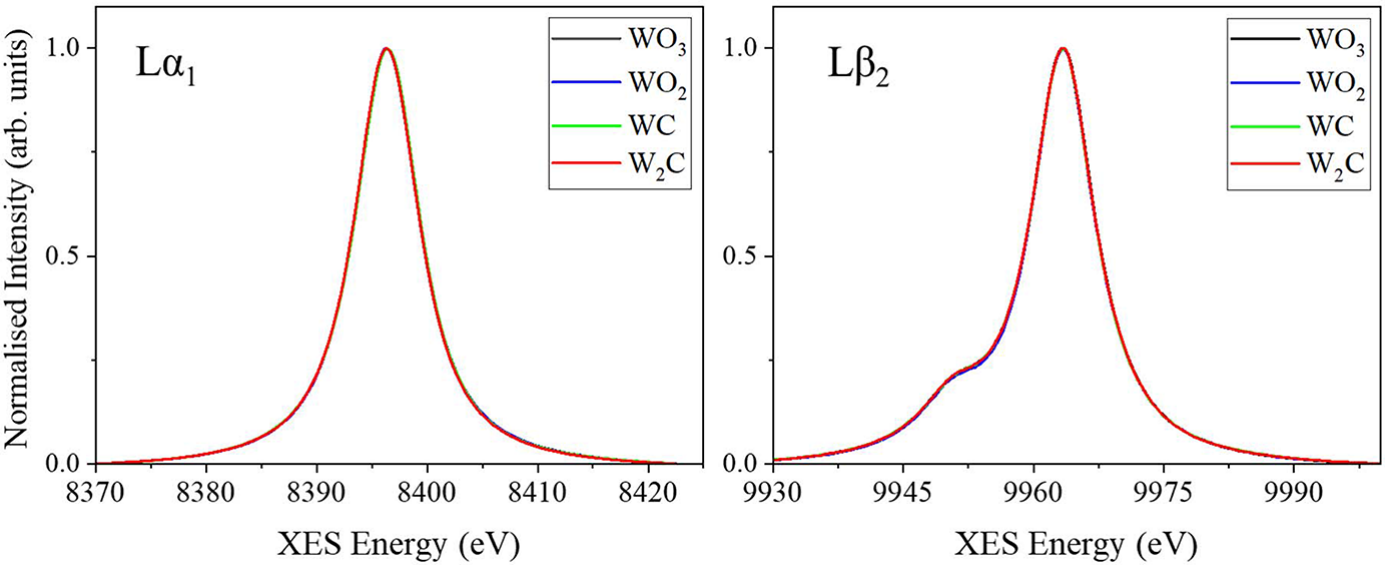
Normalized *L*α_1_ and *L*β_2_ spectra measured from WO_3_, WO_2_, WC and W2_C_ pellets in two-colour mode.

The unique design of the I20 spectrometer also allows scanning of the upper and lower rows over different energy ranges to perform two-colour XES measurements. In two-colour mode, one of the scans will define the acquisition time per data point and the number of data points, but the energy range and step size can differ for each scan. Two-colour XES spectra measured from four tungsten-containing samples are shown in Fig. 5 ▸. The incident beam was set to 10350 eV. The *L*α_1_ (3*d* → 2*p*) was measured using seven Si(444) crystal analysers on the lower row. The crystal analysers were scanned across the emission energy (*E*_f_) range between 8370 eV and 8422.5 eV using an energy step of 0.3 eV. The *L*β_2_ line (4*d* → 2*p)* was measured with seven Si(555) crystal analysers on the upper row, scanning from 9930 to 10000 eV with an energy step of 0.4 eV. In all cases the acquisition time used was 2 s per data point. The measured spectra of the different reference compounds were normalized to the maximum intensity to account for differences in tungsten concentration. The deep core-to-core emission lines from the 3*d* and 4*d* orbitals show no significant differences between the tungsten compounds we measured.

### 3.2. HERFD-XANES (XANES: X-ray absorption near-edge structure)

HERFD-XAS data are collected by aligning the spectrometer to the maximum of the emission line of interest, while the incident energy is scanned through the absorption edge (XANES) and/or over the extended energy region (EXAFS: extended X-ray absorption fine structure). The intensity variation of the emitted line is recorded as a function of the incident energy. If the energy resolution of the spectrometer is comparable with the core-hole lifetime, this can effectively reduce the lifetime broadening and improve the spectral resolution in the XANES features (Hämäläinen *et al.*, 1991 ▸; de Groot *et al.*, 2002 ▸).

**Figure 6:**
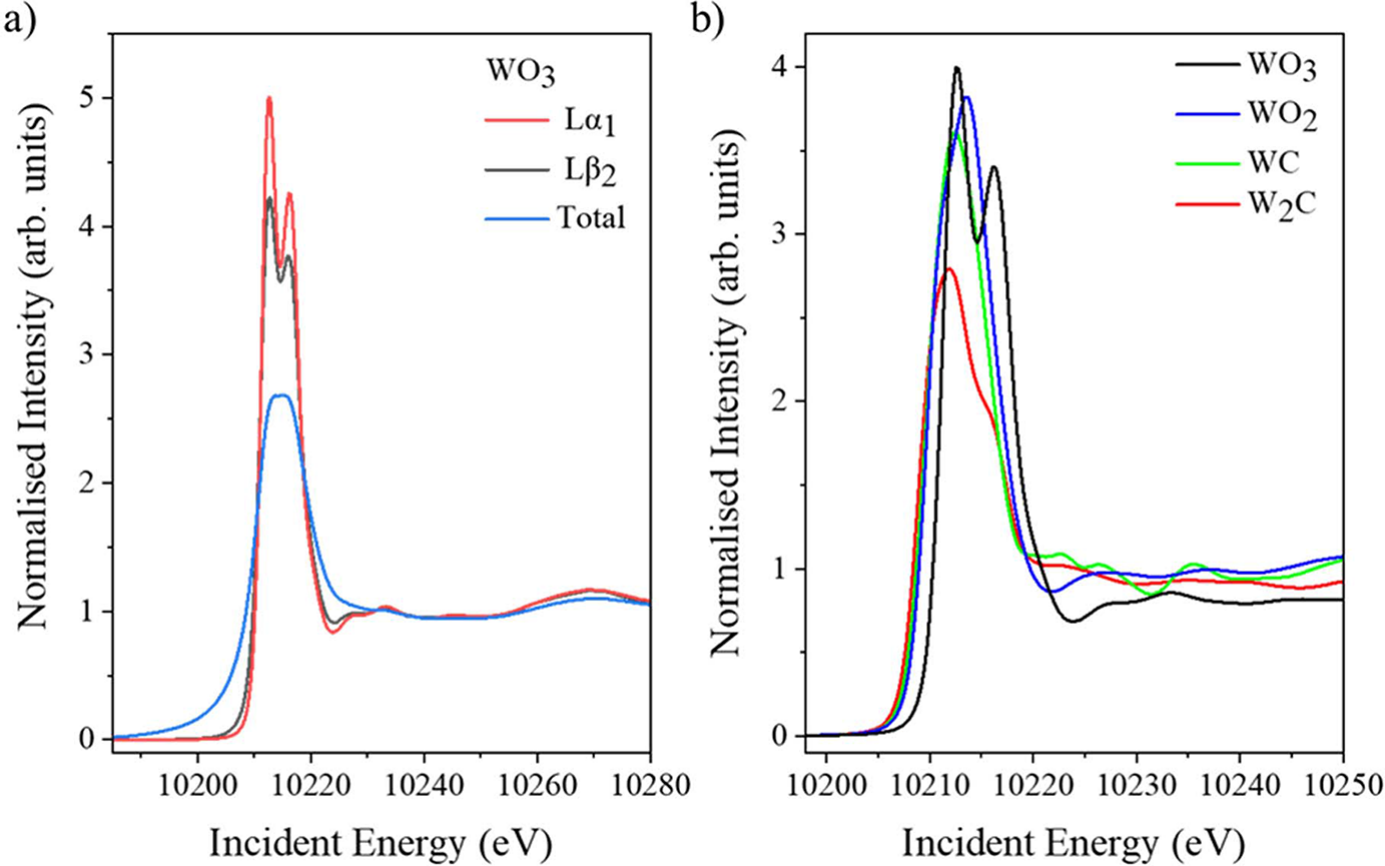
HERFD-XANES spectra measured from diluted W standard samples. The pellets are mixed with cellulose to minimize self-absorption effects. (*a*) Comparison between *L*α_1_ HERFD, *L*β_2_ HERFD and total fluorescence measured simultaneously from WO_3_. (*b*) *L*α_1_ HERFD-XANES taken from WO_3_, WO_2_, WC and W_2_C pellets. All the spectra were normalized to the edge jump.

Taking advantage of the unique capability of the I20 spectrometer, two different features or emission lines can be selected and simultaneously measure XANES from both the upper and lower rows. An example of two-colour XANES measurements is shown in Fig. 6 ▸(*a*). The *L*α_1_-XANES was measured from a diluted WO_3_ pellet with seven Si(444) crystal analysers mounted on the lower row, while the *L*β_2_-XANES was measured with seven Si(555) crystal analysers on the upper row. In both cases, the spectrometer was tuned to the peak position of the corresponding emission line measured at the incident energy of 10350 eV (Fig. 5 ▸). The spectrometer is also equipped with a four-element Vortex detector [Fig. 1 ▸(*c*)], and total fluorescence-XANES measured during the same scan has been included in the figure for comparison. The spectrometer demonstrates clear improvements in spectral resolution when compared with total fluorescence data, but the enhancement is less pronounced in the HERFD-XANES measurements taken using the *L*β_2_ emission line. The difference in resolution between the measurements on the different emission lines cannot be attributed to the instrument: the measured resolution of the spectrometer at 82.8° using Si(555) crystal analysers for the *L*β_2_ emission is ∼1.3 eV, while ∼2.0 eV is achieved with Si(444) at 70° for the *L*α_1_ emission. The measured resolution is a convolution of both the incident energy and spectrometer resolutions, with the incident energy contributing ∼1.0 eV to the overall measured value. The different resolution is instead attributed to the larger final-state lifetime broadening of the *L*β_2_ line (∼9 eV, W 4*d*) compared with the *L*α_1_ line (∼2 eV, W 3*d*) (de Groot *et al.*, 2002 ▸; Glatzel *et al.*, 2009 ▸; Ravel & Newville, 2005 ▸). The intermediate-state core-hole broadening, associated with a 2*p* core hole (W *L*_3_), is the same for both lines (∼4.6 eV). We note that a similar trend has been observed for the first-row transition metals, where it is generally recommended to use the *K*α line instead of the *K*β line, for HERFD-XANES measurements when optimal resolution and signal-to-noise ratio are required (Evans, 2018 ▸).

The W *L*_3_ XANES involves electronic transitions from the 2*p*_3/2_ core level and is known to probe the 5*d* unoccupied states to reveal ligand field splitting of the 5*d* orbitals (Yamazoe *et al.*, 2008 ▸; Jayarathne *et al.*, 2014 ▸). Compared with conventional XANES, distinct features of the white line are observed in the reference samples and show significant differences in shape and intensity [Fig. 6 ▸(*b*)]. WO_3_ exhibits a well defined double-peak structure in the white-line region, which is indicative of a pronounced ligand field splitting of the 5*d* orbitals. In contrast, WO_2_ and W_2_C display broader or less resolved features, including a subtle shoulder rather than a distinct peak separation. These different spectral features indicate a significant difference in the 5*dT*_2g_ and *e*_g_ orbital configurations and provide information on the local structure around the tungsten ion (Yamazoe *et al.*, 2008 ▸). Furthermore, HERFD-XANES reveals a sharper absorption edge and resolves multiple scattering XANES features, which are often smeared out in conventional *L*-edge spectra of heavier elements. These spectral enhancements enable qualitative and simulation analysis to extract more detailed local electronic and structural information from *L*-edge XANES spectra than with the conventional total fluorescence method.

### 3.3. Resonance XES

**Figure 7:**
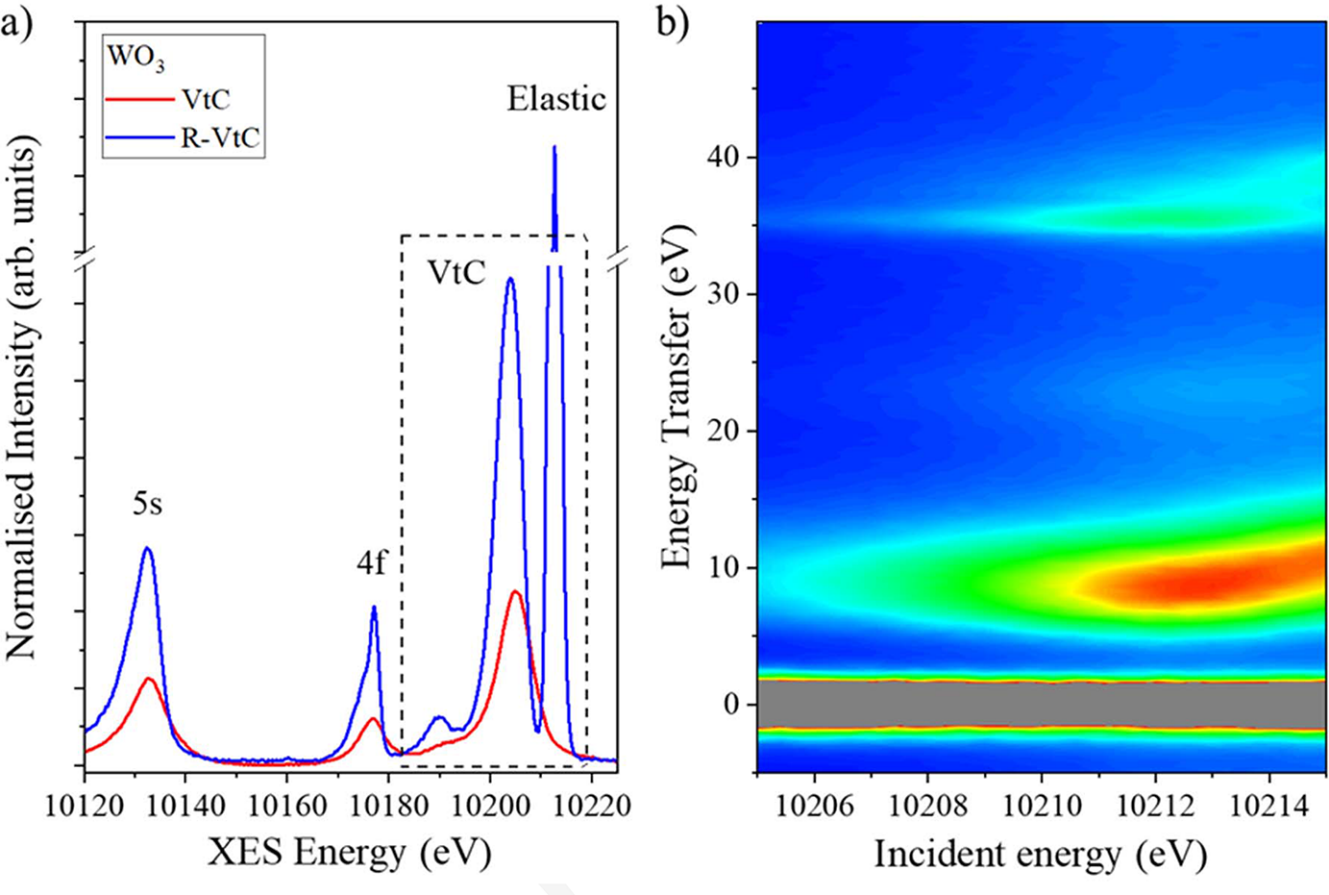
(*a*) Comparison of resonant and non-resonant VtC XES spectra taken from the same WO_3_ pellet. The spectra have been normalized and the background removed. The elastic line corresponds to the incident (excitation) energy. (*b*) RXES map collected from WO_3_ in energy-transfer mode. The map can also be taken in two-colour mode.

RXES is a technique that excites a core electron into an unoccupied state by tuning the incident energy to a specific bound state and measures the energy of emitted photons as the system relaxes. RXES measurements are therefore taken as a function of both the incident (*E*_i_) and the emitted (*E*_f_) energies across the feature of interest to locate potential resonant or excitation states that may not be visible in a non-resonant scan. Fig. 7 ▸(*a*) shows a comparison of resonant (R-VtC) and non-resonant VtC XES scans taken from WO_3_. For this R-VtC scan, the incident energy was set to the white line (10212.7 eV) to promote a 2*p* core electron to the lowest unoccupied molecular 5*d* orbital (LUMO), while the emitted energy was scanned. Although all the main features in the VtC spectrum are also present in the R-VtC spectrum, the valence features are sharper and more pronounced, thereby enhancing the amount of information that can be extracted from the data. The R-VtC spectrum is also shifted in energy, as the final state effectively probes an energy gap (*E*_i_ − *E*_f_) with an electron occupying the 5*d* LUMO, and the overall intensity is enhanced because R-VtC is measured on top of the white line. The enhancement in spectral resolution is a result of a narrower spread of final states in R-VtC, which reduces the lifetime broadening contributions (Glatzel *et al.*, 2009 ▸; Pollock & Debefve, 2023 ▸). Interestingly, we also see a significant sharpening of the 4*f* features, which suggests this feature could be useful for probing interactions between the 4*f* and valence orbitals. Further analysis of the RXES and VtC spectra from the standard tungsten samples using advanced simulation tools is on-going, and it will be reported in a future paper.

RXES measurements are typically acquired at multiple incident energies across the absorption edge, resulting in two-dimensional maps that plot emission energy against incident energy. In some cases, the data are represented as energy transfer (*E*_i_ − *E*_f_) versus incident energy (*E*_i_), and the acquisition software developed for beamline I20 supports both types of data collection. Fig. 7 ▸(*b*) shows a RXES map taken from WO_3_ in energy transfer mode with the *y* axis (*E*_i_ − *E*_f_) ranging from 50 eV to −10 eV using a step size of 0.4 eV, and the *x* axis ranging from 10205 eV to 10215 eV (*E*_i_) using a step size of 0.4 eV. Each point was acquired using a 2 s acquisition time. A slice along the constant incident energy in Fig. 7 ▸(*b*) is equivalent to an R-VtC scan. The obtained map is consistent with the previously published data and reproduces all key spectral features (Smolentsev *et al.*, 2011 ▸). Acquiring a full two-dimensional map is highly recommended as it provides a complete view of RXES and shows how excitation or resonance features are dispersed in energy. For instance, the clear presence of a gap in Fig. 7 ▸(*b*) between the elastic line (LUMO) and the VtC line confirms that WO_3_ is a non-metal. It should be noted that collecting RXES maps can take a considerable amount of time, and radiation damage needs to be mitigated when measuring sensitive samples. The RXES map shown in Fig. 7 ▸(*b*) took approximately 4 h to collect.

### 3.4. Novel scanning method: quick-scanning XES (q-XES)

One of the main limitations of the point-to-point XES spectrometers is the data acquisition speed. This is due to the need to move together a large number of motorized axes to maintain the analysers and detector in the Rowland geometry. In the case of the I20 spectrometer, over 60 axes need to move simultaneously whenever the energy change is requested. This adds about 400 ms of dead time per data point during a step scan, with the detector stages taking the longest to complete each movement.

To address this limitation, we have implemented an innovative acquisition mode called quick-scanning XES (q-XES). This method improves acquisition efficiency by reducing the number of scanned axes at the cost of deviating from the ideal Rowland geometry. Initially, the spectrometer is positioned at the centre of a specific feature, such as the peak of the emission line. At this point, the crystal analysers and detectors are still satisfying the Rowland condition, and the corrected photons arrive at the centre of the detector. From this position, only the crystal analyser pitch stages are scanned together in a coordinated motion, and the other crystal analyser and detector stages remain stationary. Coordinated motion of multiple stages is technically challenging. To realize this, we integrated *Malcolm* (Cobb *et al.*, 2013 ▸) developed at Diamond Light Source into our beamline control system. *Malcolm* is a Python-based, middle-layer framework that enables the synchronization of all pitch stages along a common continuous trajectory, while detector triggering and position feedback are processed by the PandA synchronization device (Zhang, 2016 ▸). The pitch stages can also be moved bidirectionally to reduce dead time between scans. Compared with the recently published work (Tayal *et al.*, 2024 ▸), a key advantage of our approach is that all the crystal analysers focus on the same position in the detector, and the focused spots walk vertically together along the *y* axis of the detector (see Fig. 4 ▸) as the pitch stages are scanned. This ensures the detection limit is not compromised for q-XES measurements. We typically limit the energy range of the q-XES scans to less than ±10 eV to remain close to the Rowland condition. This energy range is sufficient to capture most XES features.

**Figure 8:**
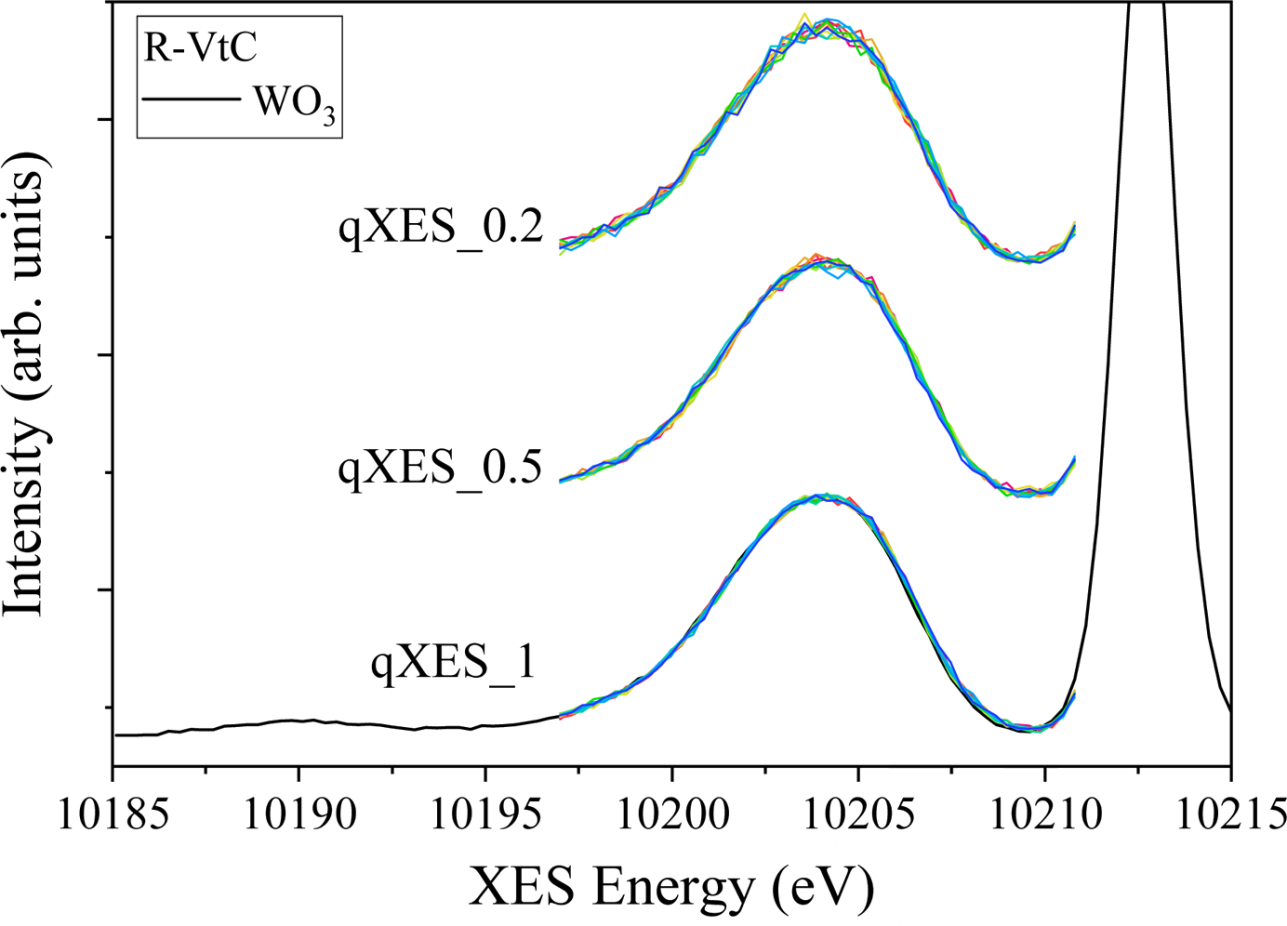
Ten bi-directional continuous q-XES spectra taken from WO_3_. q-XES_1, q-XES_0.5 and q-XES_0.2 were acquired with acquisition times of 1.0 s, 0.5 s and 0.2 s per step, respectively. For visual clarity, the qXES_0.5 and qXES_0.2 spectra have been vertically offset along the *y* axis. The black line is an equivalent scan taken in step-scan mode.

Fig. 8 ▸ shows three ten bidirectional q-XES spectra collected on WO_3_ in the VtC emission range with three different trajectory times. All scans covered the same emission energy range, from 10191.9 eV to 10205.9 eV (±7 eV). The q-XES_1, q-XES_0.5 and q-XES_0.2 scans were set up with a step size of 0.3 eV, and 1.0 s, 0.5 s and 0.2 s acquisition time per step, respectively. The total time for ten bidirectional scans for q-XES_1 was 443 s (∼49.3 s per scan), 231.5 s for q-XES_0.5 (∼25.8 s per scan) and 104.3 s for q-XES_0.2 (∼11.7 s per scan). All the q-XES scans show good agreement with the spectrum obtained in step-scan mode, with no noticeable deviations in intensity or energy with the speed of acquisition. The spectral shape is clearly reproduced with equivalent signal-to-noise despite a larger ROI being required for qXES measurements, demonstrating that even a weak feature can be collected in tens of seconds in q-XES mode. Aside from the obvious advantage of being able to perform XES in a time-resolved manner, q-XES can also be used to assess radiation damage and accumulate a better signal-to-noise ratio in specific areas of the spectrum. The implementation of q-XES helps to bridge the gap with von Hamos spectrometers while maintaining the advantages offered by the point-to-point spectrometer.

## 4. Conclusions

The successful design and implementation of the 14-crystal analyser X-ray emission spectrometer has led to significant improvements in both performance and usability of X-ray emission spectroscopy on the I20 beamline. Since operations began in 2023, the reliability of the spectrometer has steadily improved, and hardware, motion, software and control issues are now rare occurrences during user experiments. This has been a major challenge as the spectrometer is a complex instrument with over 60 motorized axes, and all the stages need to work seamlessly together to change the emission energy. The set-up process, which includes replacing the analysers, verifying the calibration and energy resolution for each row once aligned, and configuring the scan parameters, has also been made semi-automatic, allowing all 14 analysers to be changed and realigned to measure different emission lines in under 5 h. The improved detection efficiency provided by the spectrometer has enabled measurements under more realistic sample conditions of concentration and/or of weak emission lines. The user community has also welcomed the addition of the two-colour mode of operation, which is now widely used in an increasing number of experiments. Furthermore, the development of q-XES mode offers the potential for significantly faster data acquisition compared with previous point-to-point spectrometers, creating new opportunities for time-resolved studies and assessing the feasibility of the experiment in radiation-sensitive samples. The 14-analyser spectrometer is currently one of the largest hard X-ray emission spectrometers in operation worldwide, and we are confident that the instrument will continue to advance the applications of X-ray emission spectroscopy and support the growing user community.

## Data Availability

The experimental data collected at Diamond Light Source are archived in the Diamond data archive in accordance with Diamond’s Data Management policy and are available on request.
